# Home- and community-based care in the new generation of Medicaid administrative data

**DOI:** 10.1007/s10742-024-00325-6

**Published:** 2024-05-23

**Authors:** Sijiu Wang, Mingyu Qi, R. Tamara Konetzka

**Affiliations:** 1 University of Chicago, Chicago, USA; 2 Tsinghua University, Beijing, China

**Keywords:** Medicaid, HCBS, Long-term care, Administrative data

## Abstract

This study aimed to assess the quality of Home and Community-Based Services (HCBS) data elements within the Transformed Medicaid Statistical Information System Analytical File (TAF) and to devise strategies for improving its research usability. Analyzing Medicaid TAF data from 2016 to 2018, we conducted a retrospective data quality analysis, focusing specifically on HCBS-related data elements. Through secondary data analysis, we identified significant challenges, including considerable missingness and inconsistencies that hamper the effective use of TAF for research purposes. Despite these issues, we developed three approaches that enabled us to identify 94% of known 1915(c) waiver claims as HCBS. Our study also revealed considerable cross-state variations in data quality, prompting specific recommendations for utilizing HCBS data within TAF. Ultimately, the study concludes that while challenges exist, applying our recommended strategies can yield data of acceptable quality for most states, particularly in identifying HCBS usage and classifying them into service categories. Given the growing importance of home-based care, there is a pressing need to prioritize improvements in TAF’s HCBS data quality to better inform policy and practice.

**Supplementary Information:**

The online version contains supplementary material available at 10.1007/s10742-024-00325-6.

## Introduction

Medicaid administrative data are widely used by researchers to answer important questions about Medicaid policies, beneficiaries, and services. In late 2019, Center for Medicare and Medicaid Services (CMS) released a new generation of Medicaid administrative data for all states from 2016 onwards. This was exciting news for researchers, as the new data, Transformed Medicaid Statistical Information System (T-MSIS) Analytical Files (TAF), were intended to be timelier, more comprehensive, and of higher quality compared to the legacy Medicaid Analytic eXtract (MAX) data.

Medicaid data are particularly important for researchers and policymakers interested in long-term services and support (LTSS), as Medicaid is a primary payer for these services. Beneficiaries who use LTSS account for a disproportionate amount of spending in the Medicaid program. In 2020, LTSS users made up 5.4% of Medicaid beneficiaries, but accounted for 32.8% of all Medicaid expenditures.(MACPAC 2021) As a major type of LTSS, Medicaid home- and community-based services (HCBS) aims to support individuals’ preferences to remain in the community and to curb the increasing cost of Medicaid LTSS, with options like the state plan and various waivers, including the widely utilized 1915(c) waiver, which is pivotal due to its flexibility in offering tailored services. Other HCBS waiver programs, such as 1915(i), (j), and (k), while also significant, are included in the state plan and the enrollment is much smaller. Medicaid spent 95 billion dollars to provide HCBS in 2019, which accounted for 59% of total Medicaid LTSS spending. (Watts et al. 2022; Murray et al. 2021) Medicaid administrative data in its former iteration, MAX, have been used widely to investigate Medicaid HCBS, producing studies at the national and state levels, focusing on a range of research topics including access to care, quality of care, and disparities in HCBS.(Gorges, Sanghavi, and Konetzka 2019; Wang et al. 2021; Fabius et al. 2018).

For study of HCBS, the legacy MAX data offered some advantages. For years, CMS contracted with a research organization, Mathematica Policy Research, to curate the state-supplied data and transform it into a usable national data set. Mathematica researchers also developed an HCBS taxonomy to categorize claims into service types.(Peebles and Bohl 2014) The result of these efforts was that HCBS was relatively straightforward to identify and classify in MAX. Under TAF, the data are meant to be supplied in usable form directly from states, increasing the timeliness of data release and eliminating, in theory, the need for extensive transformation.

For some parts of the TAF data, including HCBS, the reality is not living up to the expectations of improved data quality. While researchers have investigated the quality of other areas of the TAF, including ambulatory care, maternal care, and hospitalizations,(Nguyen and Sanghavi 2022; Williams, Mayer, and Huser 2021; Daw et al. 2023) there is a dearth of evidence about the quality of TAF data related to HCBS, despite considerable federal and state investment in these services for low-income individuals with disabilities. Although the Data Quality (DQ) Atlas, the CMS website for TAF data quality, provides data quality assessments of HCBS waiver enrollment, assessments related to the use of HCBS are not provided.(Medicaid.gov 2023) An Urban Institute report stated that the identification of HCBS in TAF is unreliable and the HCBS taxonomy, which was commonly used in MAX to classify HCBS, was “poorly populated and largely unusable”,(Caswell, Waidmann, and Wei 2021) leaving researchers with little guidance about how to identify and classify types of HCBS in TAF data. To answer research questions related to Medicaid HCBS, researchers have begun to develop their own approaches to identify HCBS use from TAF data to answer empirical questions.(Kim et al. 2022, 2023) However, without a comprehensive examination of HCBS-related data quality in TAF and a validated strategy to improve data performance for identifying and classifying HCBS, idiosyncratic efforts by researchers may result in inaccurate measurement and incomparable or even misleading results.

This study aims to improve the understanding of Medicaid HCBS in TAF data. First, we conduct a comprehensive investigation of HCBS-related TAF data quality, examining HCBS use through Medicaid waivers and state plans and comparing it to the legacy MAX data, as HCBS identification in MAX was straightforward. Building on this comparison, we outline an approach to identify and classify HCBS claims in TAF data based on performance. Second, using our suggested approach, we describe the prevalence of HCBS use and the specific services being used.

## Method

### Data

We used Medicaid MAX and TAF data for 100% of Medicaid beneficiaries in all states for selected years: MAX personal summary (PS) file and other therapy (OT) files in 2011 and 2012 and TAF Demographics and Enrollment (DE) and OT files from 2016 to 2018. Data from the MAX to TAF transition period (2013 through 2015) were not included in this study as there were a mix of states with MAX and TAF data in each year, making quality examination complicated.^1^ The MAX PS file and TAF DE file are annual files that include demographic, eligibility, and enrollment data on beneficiaries enrolled for at least one day during the calendar year. The OT files contain fee-for-service (FFS) claims and managed care encounter records for a variety of Medicaid services, including HCBS.

### HCBS related data elements in TAF data

To identify which claims/encounter records are for HCBS, we mainly focused on three data elements that are related to HCBS (all in the OT file): type of service code (TOS), HCBS taxonomy, and the procedure code. Each of these data elements represents a potential standalone approach to identify HCBS.

TOS is a data element that contains the type of service for any claim or encounter record, including state plan and waiver claims. In MAX, the information from TOS was commonly used to identify HCBS. In TAF, the TOS has 133 service categories, including 22 categories labelled as HCBS and other categories that are not labelled as HCBS but that indicate similar services (e.g. personal care, home health, etc.). While this data element can be useful to differentiate HCBS from other services, it is directly reported by each state and states differ in how they apply the type of service codes to the same type of record. For this reason, CMS’ TAF data documentation warns about using TOS to systematically identify a specific service across different states without including information from other fields.(CMS 2020).

The HCBS taxonomy was added to MAX data in 2010 in order to better categorize waiver claims into meaningful HCBS categories,(Peebles and Bohl 2014) and soon it became the standard tool used by researchers to identify and classify HCBS. Based on procedure codes in the OT file, the taxonomy used a comprehensive (and publicly available through 2015) crosswalk to assign each 1915(c) waiver claim into 1 of 18 HCBS categories. As it was developed mainly for services provided through 1915(c) waivers, this data element is not available for most state plan claims. Unlike in MAX where HCBS taxonomy information was systematically added to the national data by CMS contractors, in TAF it is directly reported by individual states and data quality variation across states is expected. Indeed, it has been documented that in TAF the HCBS taxonomy codes reported by more than half of states were completely or mostly missing.(Caswell, Waidmann, and Wei 2021) This leads to the need for new approaches to identify and classify HCBS provided in waiver programs.

Procedure codes, mainly Current Procedural Terminology (CPT) and Healthcare Common Procedure Coding System (HCPCS) codes, provide information about the service associated with each claim. Given the data quality concerns about the HCBS taxonomy in TAF, we used procedure codes and the HCBS crosswalks to recreate taxonomy categories. HCBS crosswalks were created by Mathematica for classifying HCBS claims in MAX data into one of the HCBS taxonomy categories.(Peebles and Bohl 2014) As these crosswalks were only available if MAX data was produced for that state in the given year, the most recent national crosswalk was the 2012 crosswalk; it was available for only selected states between 2013 and 2015, and was not produced after 2016. Therefore, to obtain a relatively up-to-date crosswalk that can be used in all states, we constructed a national crosswalk by combining HCBS crosswalks published in 2012 through 2015. More specifically, we compiled the crosswalks in these four years, kept all unique mapping rules and removed duplicated ones, then we applied this combined crosswalk to TAF data from 2016 to 2018.

Other data elements that are not directly related to HCBS but were used to determine the nature of the claim include: program type code, waiver type code, claim type code, benefit type code, bill type code, and place of service. The data elements used in this study are described in Appendix Table 1.

**Table 1:** Types of HCBS in all HCBS claims/encounter records, by FFS vs. managed care (MC), and by 1915(c) wavier vs. state plan

Type of HCBS	All	FFS	MC	1915(c) waiver	state plan
Total number of claims/encounter records (million)	687	462	225	293	394
**percent of HCBS (%)**					
Home-based services	30.7	34.2	23.5	27.3	33.2
Round-the-clock services	9.0	11.9	3.0	16.4	3.5
Other mental health and behavioral services	7.3	3.0	16.3	4.8	9.2
Day services	6.3	6.8	5.3	11.7	2.3
Other services	6.3	8.9	1.0	9.1	4.2
Other health and therapeutic services	4.7	3.5	7.1	2.8	6.1
Case management	4.2	4.6	3.6	5.2	3.6
Equipment, technology, and modification	2.2	2.0	2.8	1.4	2.8
Non-medical transportation	2.2	2.8	0.8	4.3	0.6
Nursing	1.9	1.7	2.2	1.2	2.3
Home delivered meals	1.7	1.9	1.2	3.6	0.3
Caregiver support	1.6	1.4	1.9	2.3	1.0
Supported employment	1.0	1.0	0.8	1.7	0.4
Service supporting self-direction	0.8	1.1	0.1	1.7	0.1
Participant training	0.8	0.4	1.6	0.5	1.0
Expenses for live-in caregiver	< 0.1	< 0.1	0	< 0.1	< 0.1
Community transition services	< 0.1	< 0.1	< 0.1	< 0.1	< 0.1
Unknown	< 0.1	< 0.1	0	0.1	0
Missing	19.3	14.7	28.9	5.9	29.3

Note: TAF data in 2018 were used

HCBS – home and community-based services, FFS – fee-for-service, MC – managed care

### Data quality examinations and descriptive analysis

To make our investigation of the HCBS-related data quality in TAF more practically meaningful, we focus on the identification of HCBS claims from TAF and its two main applications relevant to how researchers commonly use Medicaid administrative data to study HCBS: identifying types of HCBS users, and classifying HCBS into specific service categories. For identifying HCBS claims, we first examined data missingness in HCBS-related data elements, and then used three approaches to identify HCBS from 1915(c) claims.

#### Assessing missingness in HCBS related variables

To examine missingness in key HCBS-related data elements in TAF, we examine how often the states’ TAF data have a missing value in the TOS, HCBS taxonomy, and the procedure code fields. For comparison, the same check was applied to corresponding data elements in MAX OT including CLTC (Community Long Term Care, a commonly used HCBS indicator in MAX data that was constructed based on TOS and waiver status), HCBS taxonomy, and procedure code.

#### Percent of 1915(c) claims that can be identified as HCBS

As we aim to examine the TAF data’s ability to identify HCBS from claims and encounter records, we used three distinct approaches to identify HCBS from 1915(c) claims, including the TOS approach, the HCBS taxonomy approach, and the procedure code approach. We choose 1915(c) claims instead of all Medicaid claims as the denominator, because services provided through 1915(c) are by design HCBS, and thus we can use 100% as the benchmark when we use different approaches to identify HCBS among 1915(c) waiver claims.

For TOS, as this data element includes 133 service types for a large variety of Medicaid services, the scope of HCBS needs to be determined before it can be used as an HCBS indicator. We included all 22 categories that are labelled as HCBS services, plus 14 categories where the services have a similar nature to HCBS, including home health, personal care, private duty nursing, residential care, rehabilitation, case management, hospice, and medical equipment. Consistent with prior literature, to count these as HCBS we required the place of service for private duty nursing and hospice to be at home.(Konetzka, Karon, and Potter 2012) The list of TOS codes included can be found in Appendix Table 1. TOS can identify HCBS from both Medicaid 1915(c) waiver claims and state plan claims.

For the HCBS taxonomy, the HCBS indicator was triggered if the claim was assigned to any of the 17 categories (excluding unknown) in the HCBS taxonomy data field. This indicator was only applicable to 1915(c) waiver claims and not state plan claims.

For procedure codes, we linked them to the combined 2012 to 2015 HCBS taxonomy crosswalk, following the steps specified for these crosswalks.(Mathematica 2019) If the procedure code of a claim was assigned to one of the 17 taxonomy categories (excluding the unknown category) after being linked to the crosswalk, it was identified as a HCBS claim by the procedure code approach. This approach was applied only to 1915(c) waiver claims in this study.

To assess the performance of these approaches, we applied them among 1915(c) claims and calculated the percent of claims that can be identified as HCBS by each approach. The performance is better when this rate is closer to 100%. Similar approaches were applied to MAX data for comparison.

#### Identifying type of HCBS users

The goal for this part of the investigation is to examine the usefulness of TAF for identifying different types of HCBS users, including those who use only Medicaid state plan services, only 1915(c) waiver services, or both. The state plan users were identified as those who had one or more non-waiver claims with a TOS code for HCBS and related services. The 1915(c) waiver users were identified as those with any 1915(c) waiver claims that could be identified as HCBS by any of the three approaches described in Sect. 2.3.2.

Unlike waiver claims that can be compared to internal benchmarks, no other existing data source, to the best of our knowledge, provides state-level information about the use of Medicaid state plan HCBS. This means there is no gold standard against which to assess the performance of TOS in identifying HCBS from state plan claims. We constructed the type-of-user indicator with TAF and MAX by identifying HCBS users of state plan only, waiver only, or both, and calculated the percentage of different HCBS users among all Medicaid FFS beneficiaries. In absence of a gold standard, the consistency in the composition of HCBS user type between MAX and TAF may provide some insights into TAF performance in identifying HCBS state plan users, as these calculations from MAX were generally accepted as valid.

#### Specific types of HCBS

Beyond binary HCBS use, researchers are often interested in the classification of HCBS, i.e. the ability to classify HCBS into service categories based on information from claims or encounter records. Among the state plan or 1915(c) waiver claims/encounter records that are identified as HCBS, we applied the procedure code approach and classified the identified HCBS claims into one of 17 Taxonomy categories. We described the type of HCBS for all HCBS claims, by FFS claims vs. managed care encounter records, and by state plan vs. waiver services.

## Results

### Missingness in HCBS-related variables

**Fig. 1:**
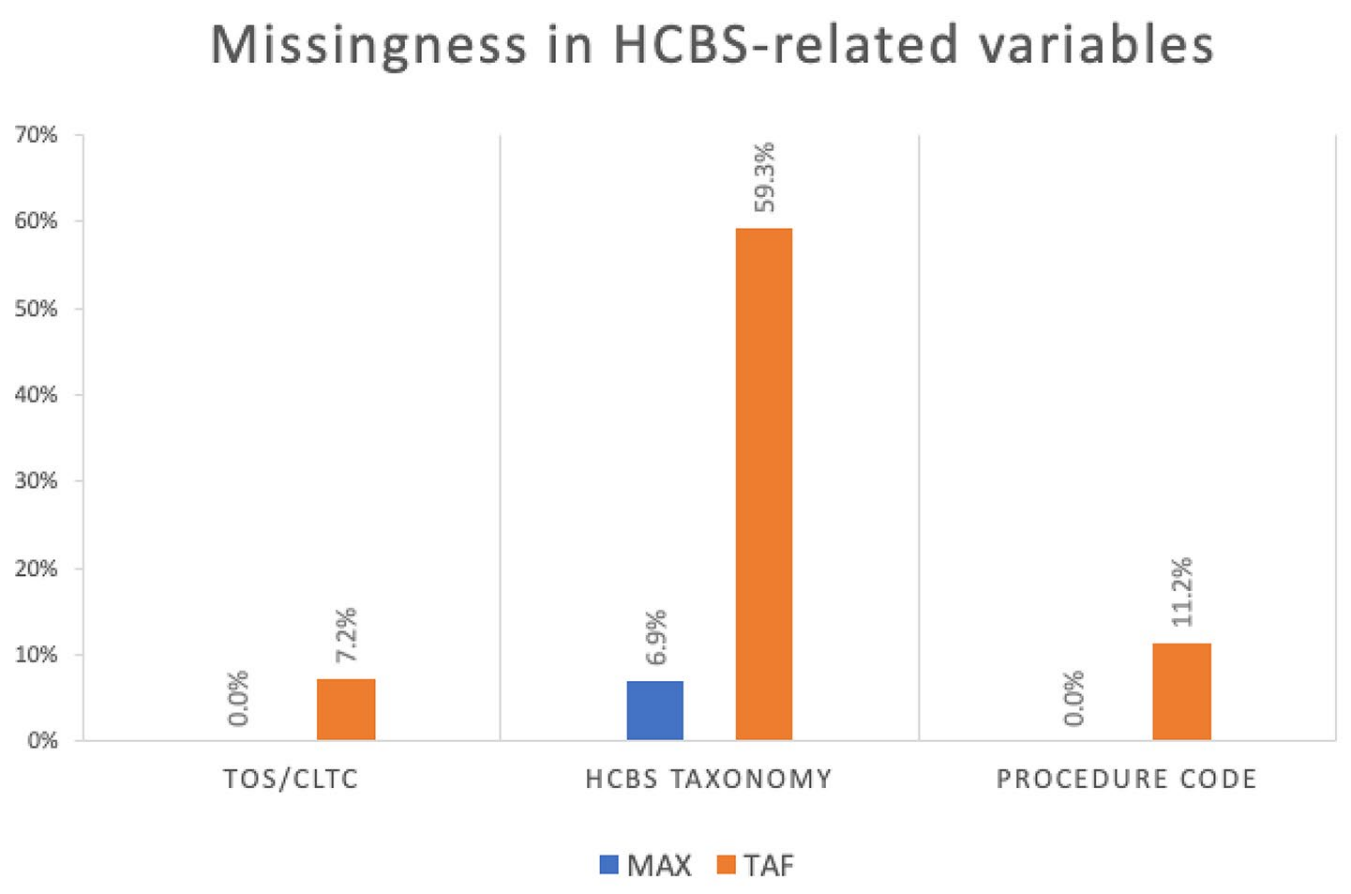
Missingness of HCBS-related variables in TAF vs. MAX. Note: HCBS – home and community-based services, TAF – T-MSIS Analytical Files, MAX - Medicaid Analytic eXtract The rate of missing for MAX included 2011 and 2012 data, and the rate of missing for TAF included 2016 to 2018 data. While the national rates are presented, the missingness varies widely across states, and the data quality in later years is not necessarily better than the earlier years

Considerable missingness was found in TAF data elements related to HCBS (Fig. 1). While in MAX data there was no missing data in CLTC and procedure codes, in TAF, 7.2% and 11.2% of 1915(c) waiver claims were missing TOS and procedure codes, respectively. The extent of missing data in the HCBS Taxonomy field is severe, with the HCBS taxonomy completely or nearly (> 95%) unpopulated for 21, 19, and 18 states in 2016, 2017, and 2018, resulting in 59.3% of missing data nationally over these three years. This makes the taxonomy field in TAF not usable for national analyses, though it may be useful for some states. In comparison, the percent of 1915(c) claims with missing taxonomy values was 6.9% in MAX data.

### Identifying HCBS among 1915(c) waiver claims

**Fig. 2:**
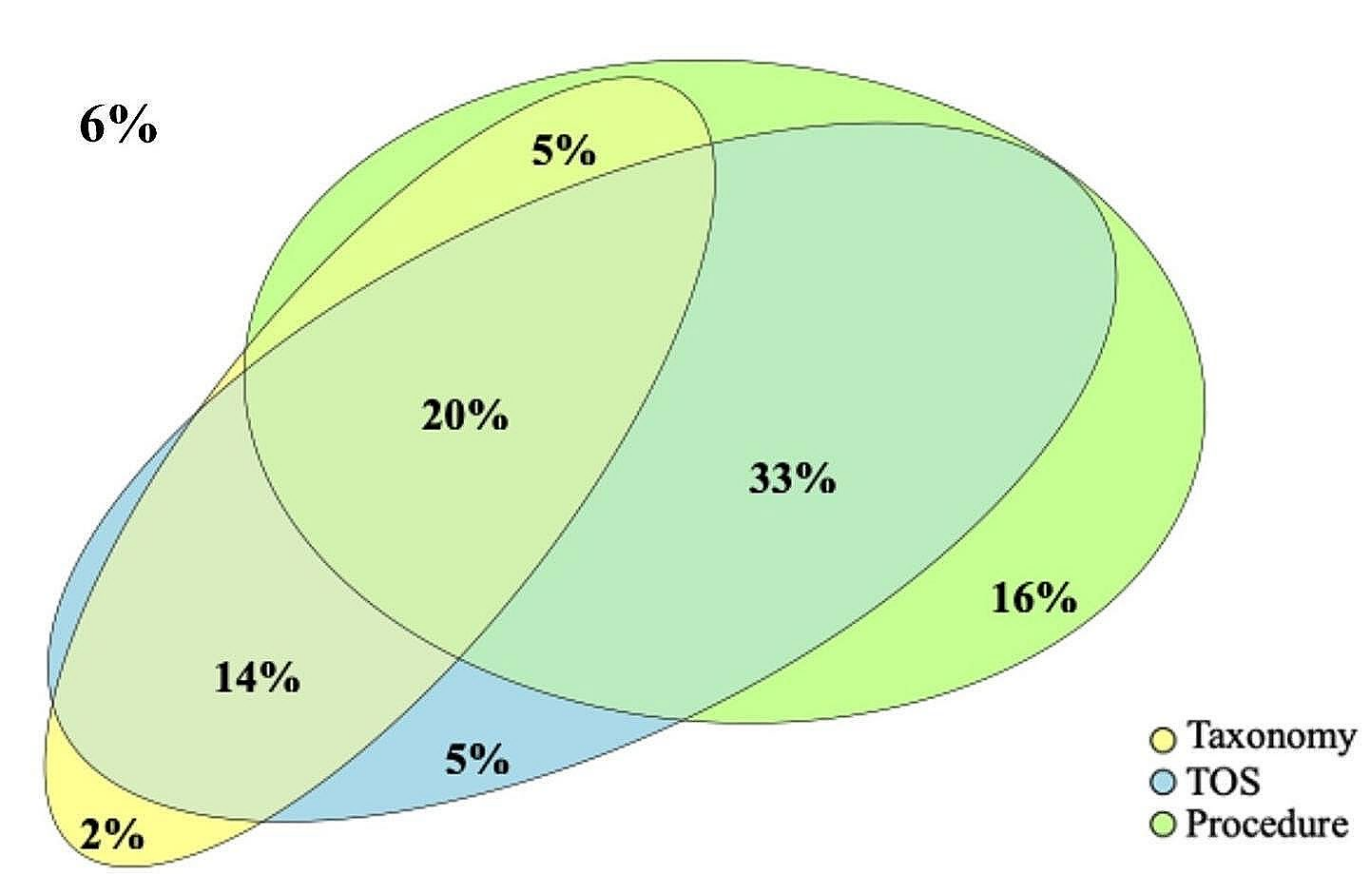
Percent of 1915(c) claims/encounter records that can be identified as HCBS with TAF data by different approaches. Note: Data source: 2016, 2017, and 2018 TAF data combined HCBS – home and community-based services. The area in the Venn Diagram is proportional to the numbers falling into each category, except for the 6% of claims/encounter records that cannot be identified by any of these three approaches.

From 2016 to 2018, 817 out of 866 million (94%) 1915(c) waiver claims could be identified as HCBS by at least one of the three approaches (TOS, HCBS taxonomy, procedure code), but only 170 million (20%) could be identified by all three (Fig. 2). HCBS claims identified by TOS only, i.e. not by the HCBS taxonomy or procedure code, were 47 million, and 18 million and 135 million were identified by the HCBS taxonomy or procedure code only, respectively. As a comparison, in 2011 and 2012 MAX data, all (> 99.9%) of the 1915(c) waiver claims were identified by at least one of these approaches, and more than 90% of claims could be identified by all three approaches, suggesting substantially higher consistency in these data elements in MAX. In addition to the data quality difference between MAX and TAF, we observed significant cross-state variation in data quality. While in 27 states all (at least 99%) 1915(c) waiver claims can be identified as HCBS by at least one of three approaches, only 35% and 54% can be identified in WA and OR, respectively.

### Types of HCBS users

**Fig. 3:**
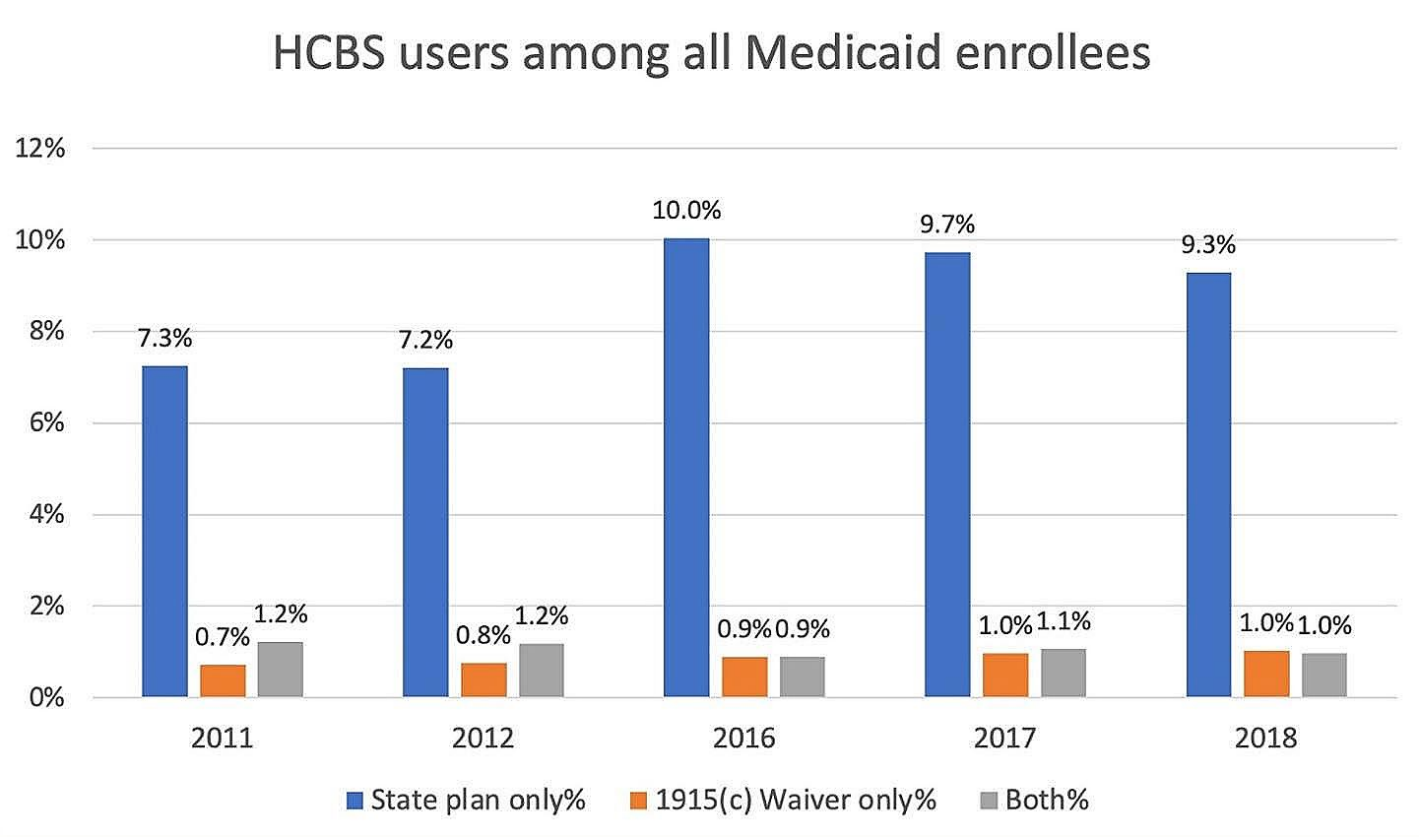
Types of HCBS users among all Medicaid enrollees by year. Note: HCBS – home and community-based services The state plan group includes all MAX or TAF OT claims/encounter records that’s not identified as 1915(c) waiver.

The types of HCBS users as a percent of total Medicaid FFS enrollees are shown in Fig. 3. For all five years included in this study, the most prevalent type of HCBS user was those who only used state plan services, ranging between 9.3 and 10.0% of all Medicaid enrollees in the years with TAF data, and around 7.2% for the years with MAX data. The prevalence of the other two types of HCBS user were similar, accounting for around 1% of Medicaid enrollees in each year.

### Specific types of HCBS

Among the HCBS claims/encounter records identified above, we were able to assign most of them to one of the 17 HCBS taxonomy categories. Using results from 2018 data as an example (Table 1), 81% of identified HCBS claims could be assigned into a taxonomy category. Thus, although the taxonomy field itself is of poor quality, the taxonomy can be re-created by researchers to some degree. The most frequently used HCBS was home-based services (30.7% of all HCBS), followed by round-the-clock services (9.0%) and mental health and behavioral services (7.3%). When comparing services provided through FFS and managed care, a higher proportion of managed care records could not be assigned to taxonomy categories (28.9%, compared to 14.7% for FFS claims). Among classified HCBS, more mental health and behavioral services and fewer round-the-clock services were provided through managed care plans, comparing to services provided on a FFS basis. When comparing services provided through 1915(c) waivers to other services, 1915(c) waivers covered more round-the-clock services and day services, while state plans provided a higher proportion of home-based services and therapeutic services. The percent of HCBS claims that cannot be classified is higher among state plan claims (29.3%) than among waiver claims (5.9%).

### Overall recommandations

To investigate research questions related to Medicaid HCBS, researchers may need to identify HCBS use from TAF data at different levels of granularity. We provide recommendations for researchers who use TAF to identify and/or classify HCBS provided through 1915(c) waiver programs or the state plan (Table 2). For research questions focusing on the use of any HCBS through 1915(c) waivers, combining all three approaches (TOS, HCBS taxonomy, procedure code) to identify HCBS waiver claims will allow the maximum use of information and will result in the broadest set of HCBS claims, while using just one or two approaches may also achieve similar rates of identification in some states (Appendix Table 2). As an alternative approach, among waiver enrollees, researchers may also consider all services provided through Medicaid 1915(c) waiver programs as HCBS, as 1915(c) waiver programs are supposed to provide solely HCBS by program design.

**Table 2:** Summary of recommended approaches to identiy and classify HCBS in TAF data

Panel 1. Identify HCBS		
**From 1915(c) waiver claims or encounter records**:		
	Use the combination of TOS, HCBS taxonomy, and procedure code approach and define claims that can be identified by any approach as HCBS. In states with very high quality data in some of the data elements related to HCBS, using one or two approach may have similar performance as using all three approaches combined. Please refer to Appendix Table 2. Alternatively, all services provided through Medicaid 1915(c) waiver programs may be considered as HCBS.	
**From non-1915(c) claims or encounter records**:		
	Use TOS approach to identify HCBS	
**Panel 2. Classify identified HCBS**		
**From 1915(c) waiver claims or encounter records**:		
	Use HCBS taxonomy first (for states with satisfactory HCBS taxonomy quality), then apply procedure code approach to claims that can’t be classified by the HCBS taxonomy	
**From non-1915(c) claims or encounter records**:		
	Use procedure code approach to classify HCBS	

Note: HCBS – home and community-based services, TAF – T-MSIS Analytical Files, TOS – type of service

For research questions focusing on specific types of HCBS, i.e. when classifying HCBS is necessary, we recommend first identifying any HCBS with the strategies recommended above, and then using a combination of the HCBS taxonomy and procedure codes to classify the identified HCBS claims/encounter records. Using the combination of the procedure code and the HCBS taxonomy can best assign HCBS claims into meaningful HCBS taxonomy categories. Another issue related to using Medicaid administrative data to identify HCBS is that researchers should be careful about interpreting the results in longitudinal studies that use both MAX and TAF data in different years, as some of the differences in results may come from different data management strategies in MAX and TAF.

## Discussion

While the new generation of Medicaid administrative data (TAF) is expected to bring data quality improvement, this is not yet the case for HCBS-related data elements. In this study, we examined TAF data quality related to HCBS data elements and identified approaches to identify HCBS from waiver claims, to identify different types of HCBS users, and to classify state plan and waiver HCBS claims into service categories. Using our recommended approach, we also described the type of HCBS users and the services being used during 2011–2012 and 2016–2018.

Several quality issues in HCBS-related TAF data were identified. First, the level of missingness in the HCBS taxonomy is high, which affects its usability in identifying HCBS and classifying waiver claims into HCBS categories. While the HCBS taxonomy is a well-developed tool and can effectively identify and categorize waiver claims from MAX data and works properly for some states with good TAF data, it cannot be used in states with a high rate of missingness or for national analyses; thus, other approaches must be adopted. Using procedure codes in conjunction with the HCBS crosswalk used to compile prior versions of the taxonomy may be a good alternative to the HCBS taxonomy fields. As the HCBS crosswalk translates procedure codes into the same HCBS taxonomy categories, it is not only useful in identifying HCBS, but is also capable of classifying HCBS and producing categories that can be compared across states.

Another issue found in TAF data is the inconsistent results produced by TOS, HCBS taxonomy, and procedure codes when identifying HCBS in waiver claims. In 2012 MAX data, 91% of claims could be identified consistently by all three approaches. This high consistency across different approaches suggests that researchers were able to choose any of these approaches and get similar results. In TAF data, however, only 20% of claims can be identified consistently by all three approaches. Meanwhile, as 23% of the claims or encounter records can be identified by only one of the three approaches, using any single approach will result in the under-identification of HCBS claims, which makes adopting a combination of multiple approaches necessary.

A long-standing concern of Medicaid administrative data is the cross-state variation in data quality, a problem in the legacy MAX data as well as the new TAF data. In our study, we observed sizable cross-state variation in the level of missingness, quality of waiver enrollment data, and the ability to identify HCBS from claims. In the HCBS taxonomy, for example, this issue is exacerbated, as populating the HCBS taxonomy shifted from being part of the national data-producing process in MAX to being reported directly by each state in TAF. Although this shift was intended to improve reporting, given that states have better knowledge about the service being provided and may be more accurate, in reality most states fail to report HCBS taxonomy codes properly, resulting in the taxonomy being unusable for many states. For data elements with low rates of missingness nationally, like TOS, state variation also exists. For example, in 2016, while the national rate of missingness in TOS among 1915(c) waiver claims was 2%, the rate of missingness was 33% in Iowa, 36% in Arkansas, and 77% in Wisconsin. In addition to missingness, another example is the ability of TOS approach to identify HCBS from waiver claims, ranging from 2% in New Jersey, 4% in Washington, to greater than 99% in twelve states.

In addition to waiver programs, Medicaid state plans are an important channel for access to HCBS, as state plan HCBS users account for a large percentage of HCBS users. Consistent with a recent report on Medicaid LTSS use, we found that state plan users account for the vast majority of HCBS users.(Kim et al. 2022) Researchers interested in identifying HCBS from Medicaid state plans may be able to use TOS in most states, a similar approach to what was used in MAX data, where the TOS data element is complete and of good quality. While we considered a broad set of services and TOS categories to be HCBS, researchers may want to make different decisions about what TOS categories to include as HCBS to fit their research questions. For those who are interested in specific types of HCBS provided through Medicaid state plans, among the HCBS claims/encounter records identified with TOS, procedure codes and the crosswalk can to applied to further classify HCBS into taxonomy categories. However, using TOS to identify specific types of HCBS is not recommended in cross-state studies, as there is no universal mapping from service to type of service code and states may apply different types of service codes to the same service.

When adopting the strategies outlined here, researchers should be aware of several caveats. First, our quality investigation and recommendations were based on 2016 to 2018 TAF data. It is possible that TAF data have improved over time, and we have observed quality improvement in some data elements in a handful of states, but the degree of HCBS-related data quality concerns were mostly consistent in all three years and exhibited no average trend toward improvement. Second, the HCBS crosswalk used in this study was originally developed for use with 2012 to 2015 MAX data. It’s possible that states’ selection of procedure codes changes over time, and newly adopted codes may have been omitted. Third, this study focused on the data quality for identifying and classifying HCBS, and more research is needed to examine other TAF data elements related to HCBS, such as the spending and amount of services. In addition, while significant improvement has been make with the approaches introduced in this study, limitations of the data still exist: The types of HCBS can only be identified with procedure codes, therefore, HCBS cannot be well classified into specific types if the procedure codes in TAF are unreliable for the state.

The recent guidance published by the CMS (CMS 2023) and the Office of the Assistant Secretary for Planning and Evaluation (ASPE 2023) on identifying and classifying HCBS in Medicaid claims offers valuable new perspectives that complement our findings. While these publications present different methodologies, they underscore the importance of the issues addressed in our research and highlight the ongoing need for innovative approaches in this area. Our study’s detailed examination of TAF data quality and the strategies we propose contribute to a growing body of knowledge that aims to enhance HCBS data analysis and application. We look forward to further supporting the advancement of data quality in Medicaid HCBS.

In conclusion, this study found that although there are quality concerns in HCBS-related TAF data, for most states the data elements to identify the use of any state plan or waiver HCBS and to classify HCBS into service categories are of acceptable quality after applying the strategies we suggest. As momentum for home-based care continues to build, data quality monitoring in HCBS-related TAF items and giving HCBS higher priority in efforts to improve TAF data quality may help improve the usefulness of TAF data to inform policy and practice in this critical area in the longer run.

## Electronic supplementary material

Below is the link to the electronic supplementary material.

Supplementary Material 1

## Footnotes

1 CMS has produced the TAF availability chart that provide the information about which states were reporting Medicaid administrative data under which protocol. The chart is available here: https://www.medicaid.gov/sites/default/files/2020-11/taf-rif-availability-chart.pdf.
