# Supplementary material for: Home- and community-based care in the new generation of Medicaid administrative data
Source: Health Serv Outcomes Res Methodol. 2024 May 23;25(1):29–41. doi: 10.1007/s10742-024-00325-6 (PMC11821789; doi:10.1007/s10742-024-00325-6)
Supplement: Supplementary file 1 — Supplementary Material 1 [file 10742_2024_325_MOESM1_ESM.docx]

**Appendix**

Appendix Table 1. Specific code values used to identify HCBS

| Approach | Data element | Values used |
| --- | --- | --- |
| TOS | TOS_CD | TOS_CD in  016, 017, 018, 019, 020, 021, 036, 043, 051, 053, 054, 062, 063, 064, 065, 066, 067, 068, 069, 070, 071, 072, 073, 074, 075, 076, 077, 078, 079, 080, 081, 082, 083, 115 |
|  | TOS_CD and POS_CD | TOS_CD = 022 or 087, and  POS_CD = 12 |
| HCBS taxonomy | HCBS_TXNMY_CD | All valid 5-digit values |
| Procedure code | LINE_PRCDR_CD  LINE_PRCDR_CD_SYS  LINE_PRCDR_MDFR_CD_1 LINE_PRCDR_MDFR_CD_2 LINE_PRCDR_MDFR_CD_3 LINE_PRCDR_MDFR_CD_4 | Procedure code was used with HCBS crosswalk, downloadable at  https://www.cms.gov/Research-Statistics-Data-and-Systems/Computer-Data-and-Systems/MedicaidDataSourcesGenInfo/MAXGeneralInformation  After downloading the file “MAX Data” for the specific year, instructions are provided in the downloaded files |

Appendix Table 2. State-by-state data quality and recommendation based on 2016-2018 TAF

| State | Quality of Approaches | | | Rate of identification | Overall Recommendation |
| --- | --- | --- | --- | --- | --- |
|  | **TOS** | **Taxonomy** | **Procedure code** |  |  |
| AK | High | High | High | >99% | Any of TOS, HCBS taxonomy, and Procedure code |
| AL | Low | Unusable | Moderate | 82% | Combined approach |
| AR | Low | Unusable | High | 99% | Procedure code |
| AZ | No 1915(c) waiver program | | | | |
| CA | Low | Unusable | High | 95% | Procedure code |
| CO | Moderate | Moderate^FN1^ | Moderate | 95% | Combined approach |
| CT | Moderate | Low | Moderate | 93% | Combined approach |
| DC | Unusable | High | Moderate | 99% | HCBS taxonomy |
| DE | High | High | High | >99% | Any of TOS, HCBS taxonomy, and Procedure code |
| FL | Low | High | High | >99% | HCBS taxonomy or Procedure code |
| GA | High | Low | High | >99% | TOS or Procedure code |
| HI^FN2^ | Unusable | Unusable | Moderate | 71% | Combined approach |
| IA | Unusable | Unusable | Moderate | 76% | Combined approach |
| ID | High | Unusable | Unusable | >99% | TOS |
| IL | High | Unusable | High | >99% | TOS or Procedure code |
| IN | High | High | High | >99% | Any of TOS, HCBS taxonomy, and Procedure code |
| KS | High | Moderate | High | >99% | TOS or Procedure code |
| KY | Unusable | High^FN3^ | High | >99% | HCBS taxonomy or Procedure code |
| LA | High | High | High | >99% | Any of TOS, HCBS taxonomy, and Procedure code |
| MA | High | Unusable^FN4^ | High | >99% | TOS or Procedure code |
| MD | High | Unusable | High | >99% | TOS or Procedure code |
| ME | Unusable | Unusable | High | 91% | Procedure code |
| MI | High | Unusable | High | >99% | TOS or Procedure code |
| MN | High | Unusable | High | >99% | TOS or Procedure code |
| MO | High | High | High | >99% | Any of TOS, HCBS taxonomy, and Procedure code |
| MS | Moderate | Moderate | Moderate | 92% | Combined approach |
| MT | High | High | High | >99% | Any of TOS, HCBS taxonomy, and Procedure code |
| NC | Unusable | Unusable | High | 97% | Procedure code |
| ND | Unusable | Unusable | Unusable | 59% | Combined approach |
| NE | Unusable | Unusable | Unusable | 75% | Combined approach |
| NH | High | High | High | >99% | Any of TOS, HCBS taxonomy, and Procedure code |
| NJ | Unusable | Unusable | High | 96% | Procedure code |
| NM | High | High | High | >99% | Any of TOS, HCBS taxonomy, and Procedure code |
| NV | Low | Unusable | Moderate | 85% | Combined approach |
| NY | Moderate | High | Unusable | >99% | HCBS taxonomy |
| OH | Unusable | Unusable | Moderate | 92% | Combined approach |
| OK | Low | High^FN5^ | High | >99% | HCBS taxonomy |
| OR | Unusable | Unusable | Unusable | 54% | Combined approach |
| PA | High | Unusable | Moderate | 93% | Combined approach |
| RI | No 1915(c) waiver program | | | | |
| SC | Low | Unusable | Moderate | 84% | Combined approach |
| SD | High | Moderate | Moderate | >99% | TOS |
| TN | High | Unusable | Unusable | >99% | TOS |
| TX | High | High | Unusable | >99% | TOS or HCBS taxonomy |
| UT | Moderate | Unusable | Unusable | >99% | Combined approach |
| VA | Moderate | Moderate | High | 96% | Combined approach^FN6^ |
| VT | High | High | Unusable | >99% | TOS or HCBS taxonomy |
| WA | Unusable | Unusable^FN7^ | Unusable | 35% | Combined approach |
| WI | Unusable | Unusable | Moderate | 89% | Combined approach |
| WV | Moderate | Unusable | High | 98% | Procedure code |
| WY | Moderate | Unusable | Moderate | 92% | Combined approach |

High: 1915(c) claim/encounter records that can be identified by this approach is >=90%;

Moderate: >=70% and <90%;

Low: >=50% and <70%;

Unusable: <50%

FN1: CO data improved significantly from 2016 to 2018. Recommendation made based on 2018 data.

FN2: No valid data in 2016

FN3: HCBS taxonomy in 2016 for KY is mostly missing. Recommendation based on 2017 and 2018 data.

FN4: HCBS taxonomy data quality in MA has significantly improved from 2016 to 2018

FN5: HCBS taxonomy in 2016 for OK is mostly missing. Recommendation based on 2017 and 2018 data.

FN6: Data quality in VA has significantly improved from 2016 to 2018. Recommendation based on 2018 data.

FN7: HCBS taxonomy data quality in WA has significantly decreased from 2016 to 2018. Recommendation based on 2017 and 2018 data.
